# Multiple Optical Sensor Fusion for Mineral Mapping of Core Samples

**DOI:** 10.3390/s20133766

**Published:** 2020-07-05

**Authors:** Behnood Rasti, Pedram Ghamisi, Peter Seidel, Sandra Lorenz, Richard Gloaguen

**Affiliations:** Helmholtz-Zentrum Dresden-Rossendorf, Helmholtz Institute Freiberg for Resource Technology, Exploration Division, 09599 Freiberg, Germany; p.ghamisi@hzdr.de (P.G.); p.seidel@hzdr.de (P.S.); s.lorenz@hzdr.de (S.L.); r.gloaguen@hzdr.de (R.G.)

**Keywords:** multi-sensor data, optical sensor, hyperspectral, hyperspectral mixed sparse and Gaussian noise reduction (HyMiNoR), spectral imaging, data fusion, feature extraction, dimensionality reduction, support vector machine (SVM), sparse and smooth low-rank analysis (SSLRA), orthogonal total variation component analysis (OTVCA), mineral exploration

## Abstract

Geological objects are characterized by a high complexity inherent to a strong compositional variability at all scales and usually unclear class boundaries. Therefore, dedicated processing schemes are required for the analysis of such data for mineralogical mapping. On the other hand, the variety of optical sensing technology reveals different data attributes and therefore multi-sensor approaches are adapted to solve such complicated mapping problems. In this paper, we devise an adapted multi-optical sensor fusion (MOSFus) workflow which takes the geological characteristics into account. The proposed processing chain exhaustively covers all relevant stages, including data acquisition, preprocessing, feature fusion, and mineralogical mapping. The concept includes (i) a spatial feature extraction based on morphological profiles on RGB data with high spatial resolution, (ii) a specific noise reduction applied on the hyperspectral data that assumes mixed sparse and Gaussian contamination, and (iii) a subsequent dimensionality reduction using a sparse and smooth low rank analysis. The feature extraction approach allows one to fuse heterogeneous data at variable resolutions, scales, and spectral ranges and improve classification substantially. The last step of the approach, an SVM classifier, is robust to unbalanced and sparse training sets and is particularly efficient with complex imaging data. We evaluate the performance of the procedure with two different multi-optical sensor datasets. The results demonstrate the superiority of this dedicated approach over common strategies.

## 1. Introduction

Mineral mapping constitutes an important tool in many geological applications and related industry fields. Mineral exploration and mining are particularly dependent on the accurate localization and characterization of target or indicator minerals at different scales of observation. Extensive sampling campaigns delivering kilometers of drill core or remote sensing data and minerals, often indistinguishable by eye, are only two of the logistical and technical challenges of traditional approaches. Automated, fast, and non-invasive imaging techniques form the forefront of today’s developments to support geologists with this arduous task.

Reflectance spectroscopy allows the rapid characterization of mineralogical samples by the analysis of reflected light over a specific wavelength range. Hyperspectral images, which are represented by hundreds of spectral channels, can be considered as a stack of several pixel vectors in which each pixel vector represents a spectrum in detail at a range of wavelengths. With the availability of such vast spectral information, hyperspectral images have been used enormously to study the interaction of electromagnetic radiation with an object or mineral of interest [1].

Hyperspectral data have been widely utilized in a variety of fields, such as remote sensing (earth observation) [2,3], food processing and inspection [4], agriculture and forestry [5], mineralogy [6,7], and medical disease diagnosis [8]. Along with the aforementioned applications in which hyperspectral imagery plays important roles, such sensors have been used intensively for mineral mapping and raw material characterization [9,10,11]. Satellite and airborne campaigns for large-scale regional mapping [12] and drillcore scanning for the characterization of underground deposits [13] are currently the most developed application-oriented fields of hyperspectral data for mineral resources. However, close-range terrestrial [14] and drone-borne measurements [15] are emerging and allow spatially and timely detailed mapping of outcrops and mines.

The range of deployed hyperspectral sensors is wide and can be grouped best by mode of acquisition (line-by-line, band-by-band, or snapshot) and spectral range covered. For mineral mapping, the most common spectral regions comprise the visible (VIS), near infrared (NIR), short-wave infrared (SWIR), and long-wave or thermal infrared (LWIR). Each spectral range is sensitive for specific groups of minerals only; e.g., visible-near-infrared (VNIR) is most indicative for iron oxides and rare earth elements, SWIR for alteration minerals, and LWIR for rock-forming minerals [16]. Spectral imaging sensors usually acquire data only within one or a subset of these spectral ranges, partly due to sensor-technical reasons, and partly to trade a part of the maximum total data volume per time to spatial detail. Commercially available sensors provide variations of this inevitable technical compromise between spatial detail, speed, and covered spectral range. The resulting individual single sensor setup defines (and limits) the number, type, and scale of the respective detectable minerals. For example, the acquisition of spectrally well-resolved data over a broader spectral range is usually accompanied by a coarse spatial resolution and slow acquisition time and vice versa. The combined interpretation of several sensor outputs provides a promising workaround to extend the detection capabilities of a single sensor, thereby returning a larger number of detectable minerals in more detail. Sensor-specific variations in spatial resolution, however, provide not only a challenge for data alignment, but also result in different mineral mixtures to be represented by one pixel. In a previous publication [17], we approached this challenge by classifying meaningful mixed mineralogical domains instead of using a conventional, direct mineral mapping approach. This allowed us to include spectrally inactive minerals into the classification process and to map domains of interest which are not characterized by one specific mineral, but by an indicative mineral mixture. In this framework, we could demonstrate a clear increase of accuracy of mineral domain classification using a multi-sensor setup compared to a single-sensor approach. The underlying pilot processing workflow, however, was still open to improvements.

In this paper, we develop an efficient and effective piece of multisensor technology to fuse diverse optical datasets with different spectral and spatial resolutions and different spectral coverage. The proposed processing chain comprehensively covers all stages, including data acquisition, preprocessing (including data preparation, co-registration, and denoising), feature fusion, and classification. To design the proposed multisensor data fusion approach, the following important factors are investigated: (1) the advancements in the vibrant fields of machine learning and signal processing are brought together to develop a reliable mineral classification and characterization methodology; (2) the advancements of the commercial hyperspectral sensors with diverse spectral coverage and spatial resolution are taken into account for raw material mapping in mineral exploration; and (3) the integrability and added value of multi-sensor datasets are evaluated to obtain an optimal condition for the investigated application. Our captured data represent a set of geological samples and consist of RGB imagery as well as five hyperspectral sensors with unique specifications in terms of sensor design, acquisition speed, spatial resolution, and spectral range. To evaluate our developed methodology, we acquired mineralogical information from scanning electron microscopy-based mineral liberation analyses (MLA) and spectral point measurements covering the complete electromagnetic spectrum in the wavelength range between 0.35 and 15.39 μm. The datasets are coregistered using automatically extracted key points. We then perform a hyperspectral mixed Gaussian and sparse noise reduction technique, HyMiNoR [18], to hierarchically remove different noise types and improve the quality of the input data. Sparse and smooth low-rank analysis (SSLRA) [19] and orthogonal total variation component analysis (OTVCA) [20] were recently proposed in the literature (see [21] for an overview) and regarded as the state-of-the-art unsupervised feature extraction approaches. OTVCA [20] was used in [17] for the classification of core samples using a fusion of multisensor images. From here on, the proposed method in [17] is referred to as OTVCA_Fus. In this paper, SSLRA [19] is used to extract informative features from the input data which are suitable for the subsequent classification task. The extracted features are then fed as input to a support vector machine classifier with radial basis function kernel (SVM-RBF) [22] to map mineralogical classes. The performance of this proposed workflow is evaluated using two-sample subsets with different characteristics: (1) with spectrally mixed, but spatially highly unbalanced classes, and (2) with spectrally mixed, but spatially balanced classes. From the methodological point of view, the proposed approach aims to solve the following challenges:

- The lack of available training samples to develop a supervised machine learning-based fusion algorithm: This problem is tackled at different stages of the proposed workflow, making it a suitable approach for ill-posed scenarios where there is no balance between the high number of spectral channels and a very limited number of training samples. For example, all preprocessing (including denoising) and feature extraction steps are unsupervised, and therefore, the algorithms do not need any training samples for being trained. In addition, our fusion approach applies both data integration and dimensionality reduction subsequently so as to make a balance between the numbers of extracted features and training samples for the subsequent supervised classification task based on SVM. Furthermore, the SVM classifier, which is the only supervised stage existing in the proposed workflow, can classify multisensor datasets using a limited number of training samples due to its effective theory in designing hyperplanes in feature space to separate between different classes of interest.
- Existence of different noise types: The received radiance at hyperspectral sensors is often degraded by several undesired phenomena, such as an acquisition setup and instrumental (sensor) noise, which dramatically downgrade the quality of the input data [23]. To increase the signal-to-noise ratio of the input data and address the effect of different noise types, we proposed to use HyMiNoR [18], which is one of the few existing mixed-noise removal approaches recently proposed in the literature.
- Hughes phenomenon (the curse of dimensionality) [24]: The imbalance between the number of spectral channels and available training samples leads to a drop in classification performance, which is inherently caused when the number of spectral channels increases while the number of training samples remains limited. The effect of this issue is magnified when we work with high-dimensional data (e.g., hyperspectral and multisensor data), as is the case in this paper. To address this problem, the approach developed in this paper represents the high-dimensional data input into a lower-dimensional subspace.

The rest of the paper is organized as follows: Section 2 is devoted to data acquisition and preprocessing. Section 3 describes, in-depth, the proposed multi-sensor methodology. Experimental results and discussions are detailed in Section 4 and Section 5, respectively. Finally, the main concluding remarks are summarized in Section 6.

## 2. Data Acquisition and Preprocessing

### 2.1. Experimental Setup and Sensor Parameters

Five hyperspectral sensors and two RGB cameras were used in the laboratory for the spectrally resolved detection of light (note that by performing calibration measurements, the reflectance in the LWIR is obtained by separating it from the self-emissivity of the sample set, i.e., the thermal emissions of the samples), enabling the characterization of selected geological samples. Employing optical imaging sensors covering a wide spectral range from 400 to 12,000 nm, we can observe manifold spectral features of electronic, vibrational, and rotational atomic transitions, which results in a unique spectroscopic description of a certain mineral class. Additional important parameters for the applicability in the scanning of geological samples, such as spatial and spectral resolution, acquisition speed, and mode of operation are evaluated by comparing different sensors operating at similar wavelength ranges. The most notable experimental settings and used sensor parameters are shown in Table 1. Due to differences in the spatial pixel sizes of the various imaging sensors, a simple co-registration by overlaying adjacent images is not possible.

**Figure 1:**
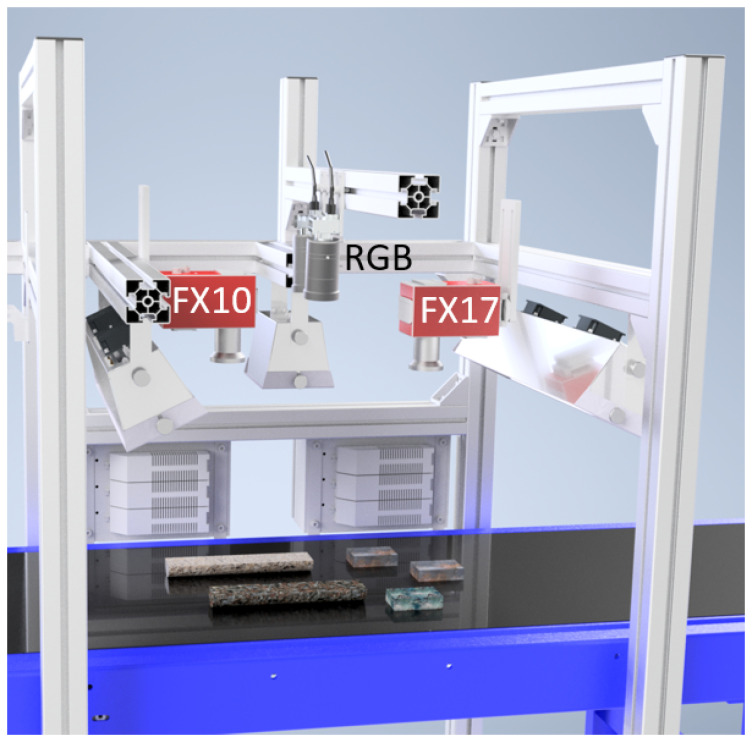
Schematic view on the set-up used for data acquisition, consisting of a multi-sensor system, which is placed above a conveyor belt. The belt has a width of 500 mm.

We employed for the reflectance spectroscopy both line-scanners and full frame-imagers, mounted in a system above a conveyor belt, whereon the geological sample set was placed (for a schematic view see Figure 1). For the detection of the reflected light in the visible-near-infrared (VNIR) and short-wave infrared (SWIR) from 400 to 2500 nm, we used four different pushbroom-scanners from Specim: the AisaFENIX (from here on FENIX), the sCMOS-50-V10E (from here on sCMOS), the FX10, and the FX17. The spectrally uniform illumination of the respective imaged area is achieved by line arrays of broad-band quartz-tungsten halogen lamps covering the VNIR and SWIR ranges. For all line scanners, the images are created over time by constant linear movement of the sample table.

The LWIR full-frame imager Hyper-Cam from Telops (from here on HC) is operated in a separate test stand in static mode; i.e., the samples are not moved during the scan. The scene in the HC setup is illuminated by two ceramic infrared quartz radiators. Due to short-time exposure of the samples to the IR radiation and negligible heating of the samples, we can omit the change in emissivities of the samples for the calculation of the reflectance. In [17], a more detailed description of the conditions of the reflectance experiments can be found. High-resolution color imaging was performed by employing two C4020 Teledyne Dalsa RGB cameras (from here on RGB) which are capable of stereo reconstruction of the observed scene.

Mineral phase analysis for validation was performed by mineral liberation analysis (MLA) with a scanning electron microscope (SEM). The MLA experiments were performed by employing a combination of an FEI Quanta 650F 169 scanning electron microscope with two Bruker Quantax X-Flash 5030 energy-dispersive X-ray 170 spectrometers and the MLA 3.1.4 software package for semi-automated data acquisition [25]. The scanning of the surface by an electron beam is combined with an automated classification of individual particles according to their X-ray emission fingerprints, resulting in spatially highly-resolved phase maps. The used pixel size was 3 μm × 3 μm and the threshold of quality for correct classification was set to 90%. Further details on the MLA experiments can be found in [13].

### 2.2. Samples Analyzed

**Figure 2:**
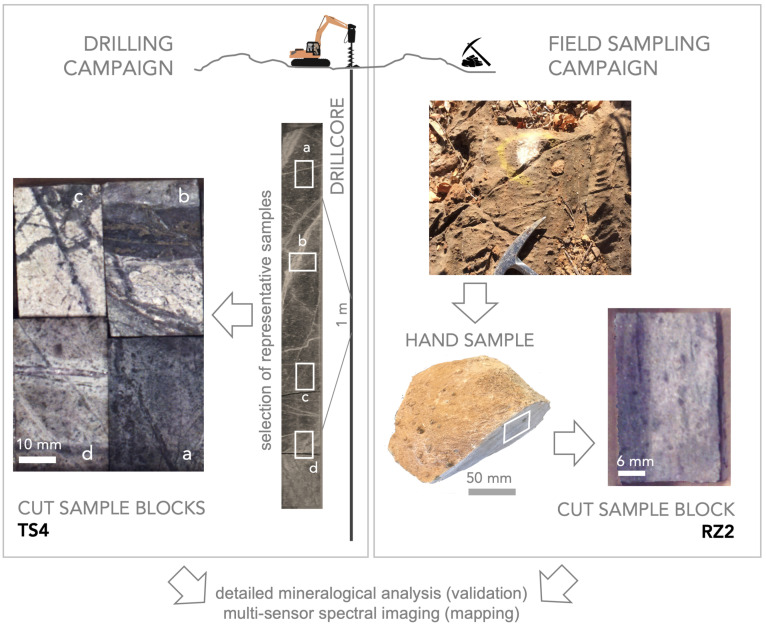
Schematic overview of the samples analyzed. (**left**) Selected representative drillcore sample blocks TS4, (**right**) sample block RZ2 from a field sampling campaign.

We investigated a set of geological samples that are relevant in current exploration projects of new mineral deposits. The clean, cut rock pieces (surface of 4 cm × 2 cm) originated from a larger hand specimen and full 1 m long drill cores, respectively (Figure 2). We selected these samples since they represent the typical lithological characteristics of mineral deposits, which we have extensively investigated for several years, e.g., by using remote sensing and near-field sensing methods [17]. Thus, we have a comprehensive understanding of the mineralogy of both sample sets, which is essential for the validation of the outcomes from the data fusion algorithm. These samples stand for two different host rocks and mineralogy. They exhibit a variety of spectral and textural features which makes them suitable to determine the abilities of our newly developed algorithm for hyperspectral reflectance data from completely different rock matrices. The first sample subset is a group of four individual rock pieces, having been cut from a drill core from the Cu-Au-porphyry deposit Bolcana, Romania (set *TS4*, samples *TS4-1551, TS4-863, TS4-1900*, and *TS4-802* shown on the left side of Figure 2). The second subset consists of a polished rock specimen from the Namibian Nb-Ta-rare earth deposit Epembe, Namibia (*RZ2* shown on the right side of Figure 2), which was collected during a field campaign on-site. All samples are fixed in a box to keep a constant spatial distance between them for the experiments with all used sensors.

MLA experiments revealed that the main occurring minerals in RZ2 are calcite, muscovite, albite, and apatite, whereas the TS4 samples contain mainly quartz, muscovite, albite, and gypsum (all in descending order) and some minerals in the few percent-range. For simplification of the mineral composition, several minerals were grouped together to create for both the RZ2 sample (albite, apatite, goethite-dominated, calcite-dominated, muscovite) and TS4 set (gypsum/anhydrite, quartz, sulfides, muscovite, feldspar) maps with five mineral classes and a background class. This background class consists of low-intensity areas where an MLA classification was not possible.

### 2.3. Preprocessing

Each dataset was preprocessed separately based on its own specific correction workflow to ensure an optimal data quality. The particular steps for each sensor are described in detail in [17] and comprise several sensor and setup-specific geometric and radiometric corrections. Stereoscopic reconstruction of the images from the two RGB cameras resulted in a 2.5D data cloud, from which an orthogonal projection to the sample plane was created and cropped to the subsets of TS4 and RZ2. The preprocessed data frames are free of spatial distortion and provide wavelength-specific reflectance information for each data pixel. For the most accurate spatial alignment, despite the differing spatial resolutions of the frames, an automated keypoint detection and matching process was applied ([17]). The aligned data was cropped to the extent of the sample to be analyzed and stacked for further analysis. Both the aligned dataset and the simplified MLA maps were resampled to match the pixel size of the sensor with the highest spatial resolution (0.08 mm). In case of the MLA maps, each resulting validation pixel was then labeled with the name of the highest abundant mineral class. Since the MLA maps were obtained from the exact sample surface, a direct validation was avoided. From the MLA map, a ground truth was defined that takes into account typical mineral mixtures or accessory minerals for each classified domain. Then, the ground truths were divided into disjoint training and test sets. In the case of TS4, the ground truths were selected from samples of the MLA results belonging to the existing visible structural regions in the RGB dataset. In the case of RZ2, from the MLA map, the most mineralogically pure pixels (about 50% of all the pixels) were selected as the ground truth.

## 3. Multi-Sensor Methodology

**Figure 3:**
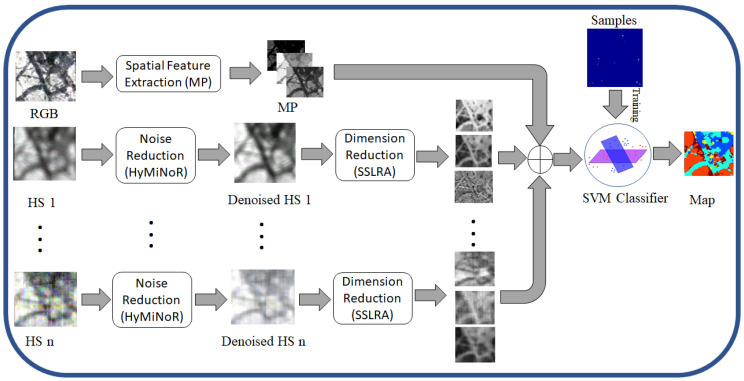
Frame-work of the proposed methodology (MOSFus) for the fusion of optical multi-sensor data for mineral mapping.

Figure 3 shows the graphical abstract of the proposed mapping methodology for optical sensor fusion. The morphological profile (MP) [26] is used to extract mineral textures from the RGB image. For hyperspectral images, first the noise and then the dimensionality are reduced using HyMiNoR and SSLRA, respectively. The reduced hyperspectral features together with the spatial features extracted from RGB are fed to the supervised classifier (i.e., SVM) to obtain the final mineral mapping. The proposed methodology consists of four main steps: 1—spatial feature extraction, 2—noise reduction, 3—dimensionality reduction, and 4—classification. These steps are explained below in detail.

### 3.1. Spatial Feature Extraction

Compared to the hyperspectral images, RGB contains higher spatial resolution and therefore can be used for spatial feature extraction. Here, the extraction of spatial information existing in the scene can be performed by spatial filters such as morphological filters based on mathematical morphology. In this work, we use MPs which are effective tools for modeling spatial information of adjacent pixels (e.g., contextual relations) in rasterized data by extracting structural features (e.g., size, geometry, etc.) [27]. MPs contain a ‘"morphological spectrum" for each pixel obtained by sequential applying of the opening/closing operations using a structuring element (SE) of varying sizes. The final profile is used for the textural analysis.

### 3.2. Noise Reduction

Hyperspectral data often contain different noise sources such as thermal, shot, and sparse noises. Hyperspectral noise reduction is a challenging task and can improve the quality of low signal-to-noise ratio images [23]. Here, we utilize a recent technique for the mixed noise reduction in hyperspectral data (HyMiNoR [18]). HyMiNoR assumes that the signal is contaminated by Gaussian noise and sparse noise and then removes the mixed noise in two steps. The hyperspectral data are first modeled by

(1) H=Y+N,

where H is a matrix containing the observed data, Y represents Gaussian noise free signal, and N is the Gaussian noise. HyMiNoR exploits an automatic technique called hyperspectral restoration (HyRes) [28] to estimate Y, and therefore we can show

(2) $$\hat{Y}=\mathrm{HyRes}\left(H\right).$$

In the case of mixed sparse and Gaussian noise, $\hat{Y}$ might contain sparse noise. Therefore, HyMiNoR assumes that

(3) $$\hat{Y}=X+S,$$

where X is the unknown (Gaussian and sparse) noise free signal and S is the sparse noise. In order to estimate X, since S is sparse, HyMiNoR uses the following minimization problem:

(4) $$J_{1}\left(X\right)=\min_{X}\;{∥\hat{Y}-X∥}_{1}+\lambda {∥DX∥}_{1},$$

where $J_{1}$ and λ are the cost function and the tuning parameter, respectively, and D denotes the first order difference matrix. An iterative algorithm was given in [18] to solve Equation (4) utilizing the alternative direction method of multipliers (ADMM) [29]. After splitting the variables using the split Bregman method and exploiting the penalty method [30] we rewrite Equation (4) as

(5) $$\arg\min_{X,V_{1},V_{2}}\;{∥V_{1}∥}_{1}+\lambda {∥V_{2}∥}_{1}+\frac{\mu_{1}}{2}{∥V_{1}-\hat{Y}+X-L_{1}∥}_{F}^{2}+\frac{\mu_{2}}{2}{∥V_{2}-DX-L_{2}∥}_{F}^{2},$$

where $L_{1}$ and $L_{2}$ are Lagrange multipliers. In [18], Equation (4) was solved by utilizing a cyclic descent (CD) algorithm [31,32]. In other words, the problem was solved with respect to one matrix at a time while the other one is assumed to be constant. The solution with respect to X is given by

(6) $$\begin{array}{cc} \; & \mathrm{SVD}\left(\mu_{2}D^{T}D\right)=US_{\mu_{2}}U^{T} \end{array}$$

(7) $$\begin{array}{c} \Lambda^{-1}=U{\left(\mu_{1}I_{p}+S_{\mu_{2}}\right)}^{-1}U^{T} \end{array}$$

(8) $$\begin{array}{c} X^{m+1}=\Lambda^{-1}\left[-\mu_{1}\left(V_{1}-Y-L_{1}\right)+\mu_{2}D^{T}\left(V_{2}-L_{2}\right)\right]. \end{array}$$

The solutions with respect to $V_{1}$ and $V_{2}$ are given by

(9) $$\begin{array}{cc} \; & V_{1}^{m+1}=\mathrm{soft}\left(\hat{Y}-X^{m+1}+L_{1},{\frac{1}{\mu }}_{1}\right) \end{array}$$

(10) $$\begin{array}{c} V_{2}^{m+1}=\mathrm{soft}\left(DX^{m+1}+L_{2},{\frac{\lambda }{\mu }}_{2}\right), \end{array}$$

where the soft function is given by

(11) $$\mathrm{soft}\left(B,a\right)=\max\left(0,\left|B\right|-a\right)\frac{B}{\left|B\right|}.$$

The final step is to update the Lagrangian multipliers,

(12) $$\begin{array}{cc} \; & L_{1}^{m+1}=L_{1}^{m}+\hat{Y}-X^{m+1}-V_{1}^{m+1}, \end{array}$$

(13) $$\begin{array}{c} L_{2}^{m+1}=L_{2}^{m}+DX^{m+1}-V_{2}^{m+1}. \end{array}$$

### 3.3. Dimensionality Reduction

Spectral signals generated using hyperspectral sensors contain redundant information. It has been shown that a hyperspectral set can be represented in a much lower dimensional space (is often called as subspace) than the sensor’s dimension [33]. However, the dimension (rank) of the subspace is unknown, and therefore, needs to be estimated [34]. Additionally, in the classification applications, which represent the aim of this paper, it has been shown that increasing the dimensionality, where the number of training samples is limited, degrades the classification performance [35]. This is known as the Hughes phenomenon [24]. Hyperspectral data used in this paper are obtained by five different sensors and therefore the dimension reduction step is a crucial step in our application. Here, we use a recently developed dimension reduction technique for hyperspectral images called sparse and smooth low rank analysis (SSLRA) [19]. SSLRA utilizes a low rank model to represent the signal with a few informative features, which is given by

(14) $$X=\left(F+S\right)V^{T}+N,$$

where F and S represent the unknown smooth and sparse components, respectively, and V contains the unknown subspace basis. To simultaneously estimate F, S, and V in Equation (14), SSLRA uses a constrained penalized cost function (CPCF) by solving

(15) $$\min_{F,S,V}\;J_{2}\left(F,S,V\right)=\min_{F,S,V}\;\frac{1}{2}{∥X-\left(F+S\right)V^{T}∥}_{F}^{2}+\lambda_{1}{∥F∥}_{\mathrm{TV}}+\lambda_{2}{∥S∥}_{1}\;\;\;s.t.\;\;\;V^{T}V=I.$$

In Equation (15), the TV-norm and the $\ell_{1}$ norm promote piecewise smoothness and sparsity on F and S, respectively. Note that the constraint $V^{T}V=I$ is exploited to enforce the orthogonality condition on the subspace and the isotropic TV penalty [30] is applied spatially on the spectral bands. SSLRA solves Equation (15) using a CD algorithm. The solution with respect to to F is given by

(16) $$\begin{array}{cc} \; & G=XV^{m}, \end{array}$$

(17) $$\begin{array}{c} F^{m+1}=\mathrm{SplitBregman}\left(G-S^{m},\lambda_{1}\right); \end{array}$$

the SplitBregman method is discussed in detail in [30]. The solution with respect to to S is given by

(18) $${\hat{S}}^{m+1}=\mathrm{soft}\left(G-F^{m+1},\lambda_{2}\right).$$

Finally, (15) is solved with respect to V as

(19) $$\begin{array}{cc} \; & \mathrm{SVD}\left(X^{T}\left(F^{m+1}+S^{m+1}\right)\right)=P{\Sigma Q}^{T}, \end{array}$$

(20) $$\begin{array}{c} V^{m+1}=PQ^{T}. \end{array}$$

The SSLRA algorithm was explained in detail in [19]. It should also be noted that the smooth features (F) extracted by SSLRA are used for classification. In this paper, SVM is utilized for the classification step, which will be discussed in the next section.

### 3.4. Classification

SVM is a widely-used approach for hyperspectral data classification due to its effective capability in handling ill-posed situations where there is no balance between the number of bands and training samples. Here, SVM is selected as the spectral classifier due to its efficiency, stability, and high accuracy compared to several widely-used machine learning-based classification techniques discussed in [36]. As shown in [36], the subsequent use of SVM on extracted features provides an accurate machine learning approach, in particular, for the classification of optical images [36].

SVM separates two classes using hyperplanes in the multidimensional feature space. In more detail, SVM searches for hyperplanes which maximize the margin from the closest training samples (called support vectors) of two classes. SVM was originally introduced to deal with linear classification problems. Since classification problems are often nonlinear, kernel tricks can be exploited to project the data into a higher dimensional feature space where the data are linearly separable. In this paper, the Gaussian radial basis function (RBF) was used as the kernel function due to its successful performance in the case of high dimensional spectral features [22,36,37]. Cross-validation is often used as the parameter selection technique to find the optimum tuning parameters, including the penalty parameters and the spread of the RBF kernel [38].

## 4. Experimental Results

### 4.1. Parameter Setting

In the experiments, the parameters for the proposed algorithm were set as follows: For the MP, a disk-shaped SE was selected with the radius sizes of 20, 50, 100, and 200 such that the morphological profile contained 27 features (12 opening, 12 closing, and the input RGB image). For HyMiNoR, the tuning parameter was selected as λ=1 while the default value used in [18] was λ=10, as we found λ=1 more suitable for the close-range imagery compared to remote sensing images used in [18]. The augmented penalty parameters $\mu_{1}=\mu_{2}=0.5$, which are the default values utilized in [18]. For SSLRA, the tuning parameters were set as $\lambda_{1}=\lambda_{2}=0.05$ (default values suggested by [19]) and the number of features for the hyperspectral images was set to r=10, and therefore, the final number of features that were fed to SVM was 77 (27 from RGB plus 50 from hyperspectral sensors). For the RBF kernel, the optimal hyperplane penalty parameters, *C*, and the spread of the RBF kernel, γ, were selected in the range of $C=10^{-2},10^{-1},...,10^{4}$ and $\gamma =10^{-3},10^{-2},...,10^{4}$, respectively, using five-fold cross validation.

### 4.2. Results: Dataset TS4

#### 4.2.1. Fusion Performance

Table 2 reports the numbers of training and test samples utilized for each mineral class. Table 3 compares the mineral mapping accuracy obtained by applying SVM on different sensors and techniques. The highest accuracy in each row is shown in bold typeface and the number of features used for the classification task is given in brackets.

**Figure 4:**
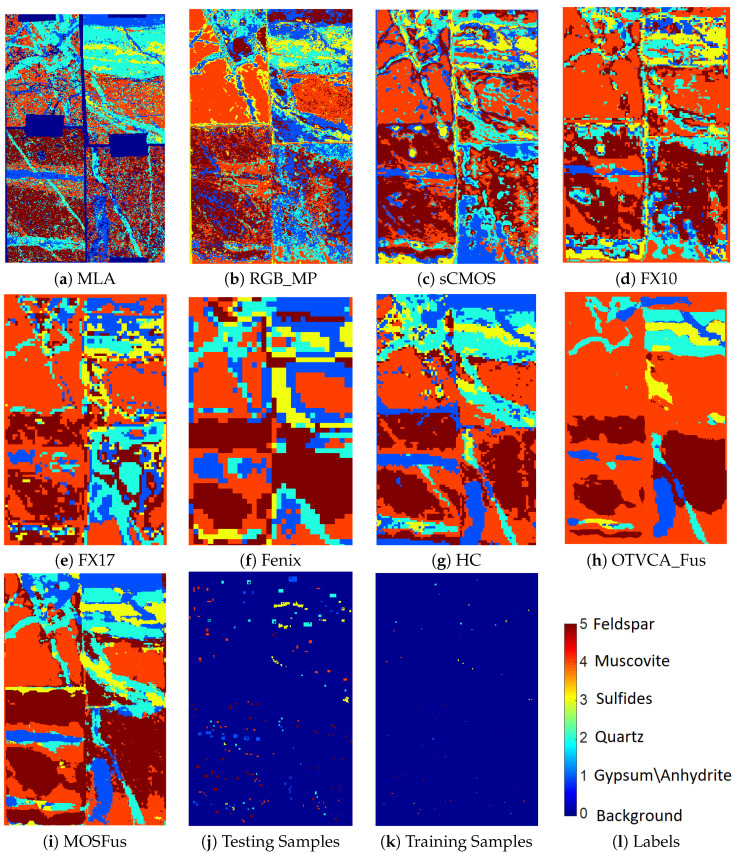
The classification maps of different fusion techniques and different sensors using SVM applied on the TS4 dataset.

The comparison reveals the advantage of the proposed fusion approach (entitled as MOSFus hereafter) compared to the other techniques in terms of average accuracy (AA), which is the mean value of the class accuracies, and the overall accuracy (OA), which is the ratio of correctly labeled samples over the entire test samples. In more detail, MOSFus with 89.53% OA provides 4.33% higher OA than the previously developed fusion method (OTVCA_Fus [17]) and 7.98% more than the highest OA obtained by a single sensor (i.e., HC). Additionally, for all classes except muscovite, MOSFus outperforms the other techniques in terms of class accuracy (CA). RGB gives the lowest OA due to the existence of limited spectral bands in such datasets. However, RGB_MP improves the OA of RGB by 11%, which reveals the advantage of including spatial information for mineral mapping. Other highlights of the results are the high accuracies obtained by HC, which will be discussed in the next subsection and in Section 5. Figure 4 depicts the mapping results of different techniques using SVM. The test and training samples used for the classifier together with the map obtained from MLA are also shown for the sake of comparison. Through visual comparison, it can be observed that MOSFus provides the best mapping in terms of structural preservation and mineral target detection. This can be further confirmed by comparing it with the highly detailed MLA mapping. We should note that although the background is labeled in the final mapping, it does not affect the accuracy assessment. Only the test samples shown affect the final classification accuracies.

#### 4.2.2. Single Sensor Performance

**Figure 5:**
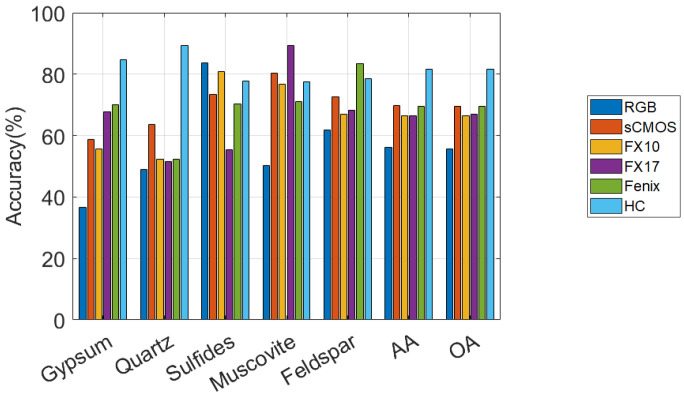
The performances of different optical sensors on different minerals in terms of accuracy in percentage obtained by applying SVM on the spectral bands of the TS4 dataset.

Figure 5 shows the comparisons of the accuracies (in percents) obtained by applying SVM on the spectral bands of the optical sensors for TS4. The comparison reveals the sensitivity of the sensors to the unique minerals. The outcomes of the experiments can be itemized as follows.

- RGB provides the lowest class accuracy compared to the other sensors due to the absence of the detailed spectra which distinguish the minerals. However, it is of interest that for sulfides, RGB provides the highest accuracy (83.63%).
- sCMOS, FX10, and FX17 perform similarly and demonstrate medium CAs for all classes compared to the other sensors. FX17 gives the highest accuracies among the other sensors for muscovite and the lowest one for sulfides. On the other hand, FX10 gives a very high CA for sulfides and performs slightly better for this class than the sCMOS which covers the same wavelength range. This could be attributed to the higher spatial resolution of the sCMOS which leads to a higher number of sulfide pixels being localized in veins where no sulfide domains were seen by the MLA validation.
- Fenix gives the highest accuracies for the Feldspar. However, this could be due to the low spatial resolution of the HSI and over classifying the pixels which can be seen from Figure 4 by comparing the mapping results of Fenix with MLA.
- HC provides very high accuracies for both quartz and gypsum and relatively good accuracies for the other three mineral classes compared to the other sensors. Additionally, the single HC sensor gives the OA of 81.55% (also see Table 3) which is considerable compared to the fusion techniques OTVCA_fus (85.27%) and MOSFus (89.57%). As a result, HC can be considered as the most suitable sensor for the minerals existing in TS4.

### 4.3. Results Dataset RZ2

#### 4.3.1. Fusion Performance

**Figure 6:**
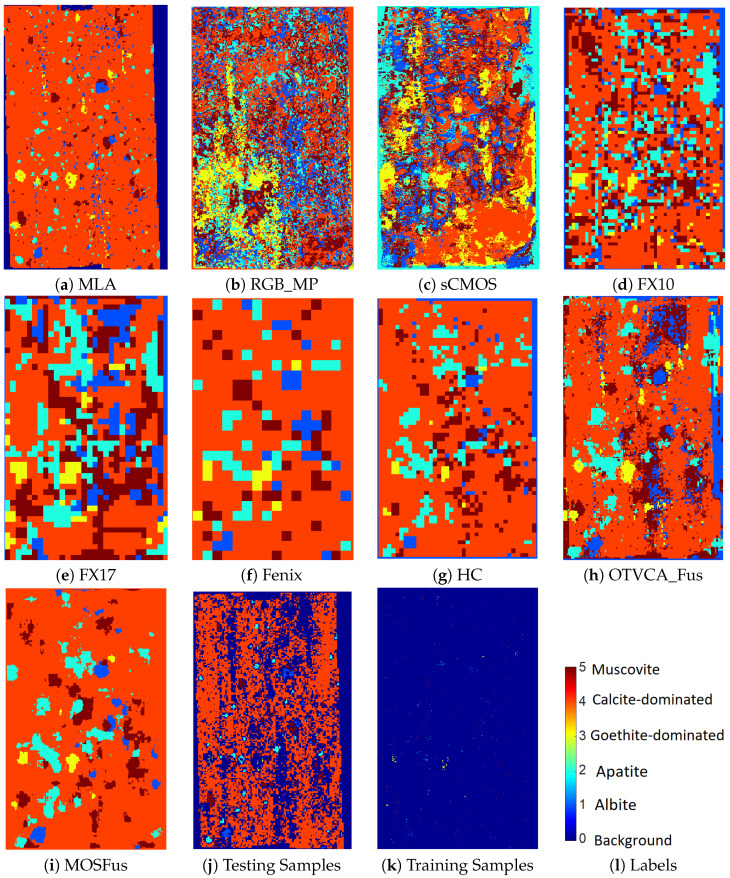
The classification maps of different fusion techniques and different sensors using SVM applied on the RZ2 dataset.

Table 4 gives the numbers of training and test samples for the mineral classes used for classification of the RZ2 sample. The classification results for the RZ2 dataset are given in Table 5. MOSFus gives OA = 86.31% and improves the one obtained by OTVCA_Fus by 8.32%. More importantly, MOSFus outperforms all the other methods in terms of class accuracies given in the table except for apatite and goethite. Similarly to TS4, the gain achieved using MP is almost 10% higher in terms of OA than the one obtained from the RGB image, which once again confirms the advantage of extracting spatial information for mineral mapping. From Table 5, we observed that HC achieves 4.38% higher OA than OTVCA_Fus; however, the AA is 0.71% lower. This can be explained by the unbalanced number of samples per class. As can be seen from Table 4, the number of test samples is imbalanced, particularly for class 4 where HC performs better than OTVCA_Fus. The large number of samples in this class can dominantly affect the OA; however, the AA shows that the performance of OTVCA_Fus is better for most of the classes. The mineral mappings obtained by applying SVM on different techniques are given in Figure 6 in addition to the MLA map and the training and test sets. The visual assessment confirms that MOSFus provides a better mapping quality compared with the other techniques both in terms of structures and resemblance with MLA. However, OTVCA_Fus also provides a decent mineral mapping compared to MOSFus. For instance, it better detects albite in the upper-right corner of the sample compared to MOSFus, which misses those samples.

#### 4.3.2. Single Sensor Performance

**Figure 7:**
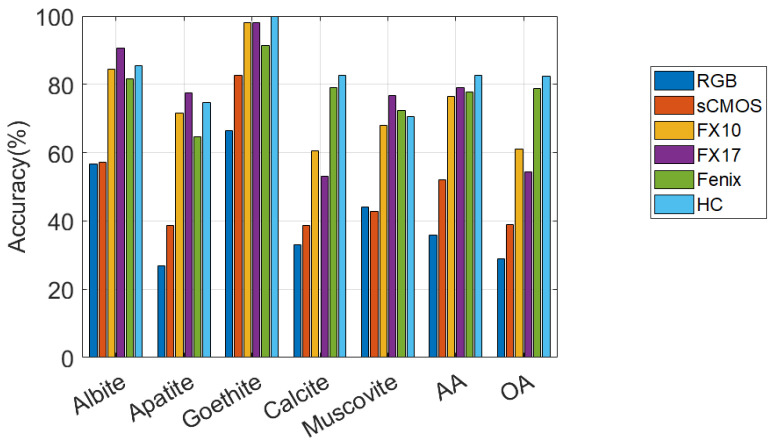
The performances of different optical sensors on different minerals in terms of accuracy in percentages obtained by applying SVM on the spectral bands of the RZ2 dataset.

Figure 7 illustrates the accuracies obtained by applying SVM on spectral bands of the individual optical sensors for RZ2 data. The outcomes of the experiments are summarized as follows.

- RGB and sCMOS perform similarly and show the lowest accuracies for all classes. However, in terms of the accuracies reported, one can conclude that RGB is the poorest sensor in terms of targeting the minerals of this dataset.
- FX10 and FX17 perform similarly and demonstrate moderate accuracies for all classes compared to the other sensors.
- Fenix also performs moderately for all the mineral targets except calcite, which it gives the high accuracy of 79.1% compared to RGB, sCMOS, FX10, and FX17.
- HC gives the highest CAs for goethite and calcite. Similarly to TS4, HC provides very high accuracies for all the classes compared with the other sensors, and by far it is considered as the most suitable sensor for targeting the minerals existing in RZ2. This could also be seen from Table 5 where the single HC sensor leads to the OA of 82.32%, which is considerably higher than OTVCA_fus (77.94%). It is also considerable given the highest OA obtained by MOSFus (86.31%).

## 5. Discussion

### 5.1. The Spectral Behaviors of Different Mineral Classes for Different Sensors

In this subsection, we provide a critical discussion of the results obtained in the experiments. It should be noted that all the discussion provided in this paper for the evaluation of the single sensor performance is from the view point of a multisensor data fusion scheme. Therefore, the spatial resolutions of different sensors and the corresponding training and test samples were resampled to be of similar pixel size. The considerable improvements in terms of classification accuracies obtained by MOSFus compared to the results obtained from the individual optical sensors further revealed the advantage of the multi-optical sensor fusion technique. Additionally, the results presented in the paper confirmed that for both datasets, the proposed fusion technique called MOSFus outperforms other techniques, particularly the recently developed method in [17]. An important advantage of MOSFus compared to OTVCA_Fus [17] is that in MOSFus the informative features are all extracted in an automatic manner, and therefore, it is considered as a framework which does not rely on the interpretation of experts in geology. On the other hand, in OTVCA_Fus, the features are selected manually and through visual conception, which is time-demanding and highly subjective.

**Figure 8:**
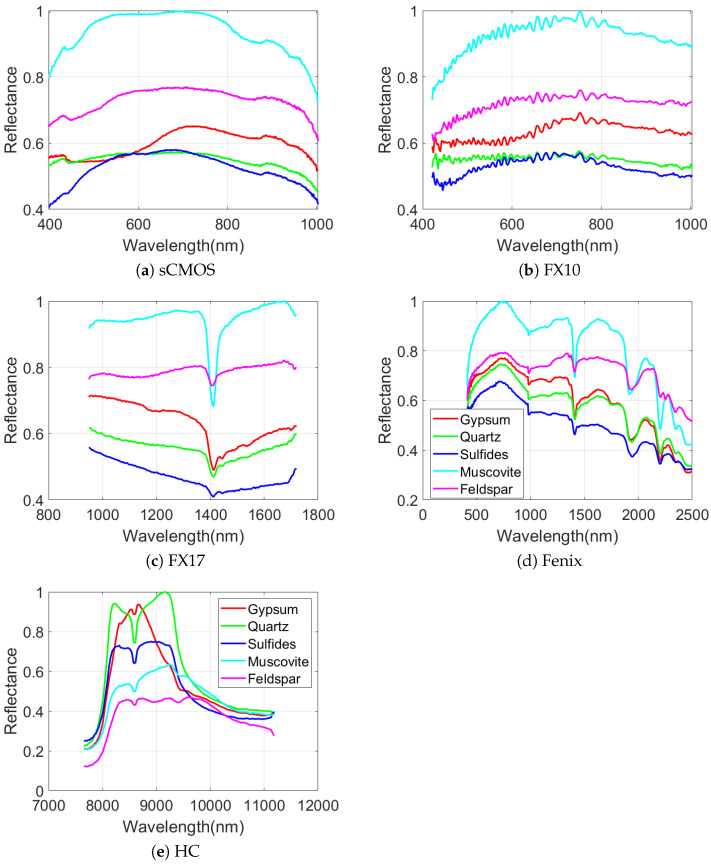
The spectral behaviors of different mineral classes of the TS4 dataset for different hyperspectral sensors. The results are based on spectral means over the minerals’ labels from the training set.

**Figure 9:**
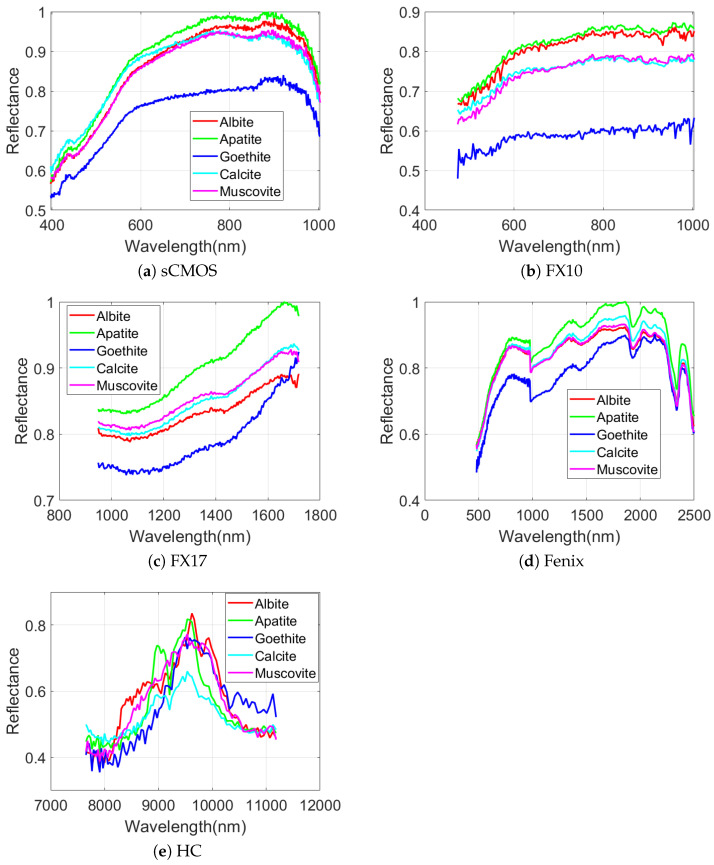
The spectral behaviors of different mineral classes of the RZ2 dataset for different hyperspectral sensors. The results are based on spectral means over the minerals’ labels from the training set.

As can be seen in Figure 5 and Figure 7, the performances of different sensors vary for different minerals for both datasets. For example, the spectra of the sCMOS and the FX10 cover the same wavelength range but show different spectral features. This can be explained by the higher sensitivity and the higher spatial resolution of the sCMOS sensor, which leads to less mixed spectra. Thus, the sCMOS exhibits more pronounced spectral features related to a single mineral class. These differences truly justify the concept of this research, i.e., developing a multi-sensor fusion technique for mineral mapping. Additionally, in terms of single sensor comparison and based on the accuracies reported for TS4 and RZ2, HC, which contains only 91 spectral bands, considerably outperforms the other sensors that contain many more spectral bands. This reveals that the spectral range of HC (i.e., LWIR) is more suitable to distinguish the mineral-domains existing in TS4 and RZ2. This can be further observed from the spectral similarity of the mineral classes. Figure 8 and Figure 9 show the mean spectral signatures for different classes of minerals existing in the training sets for TS4 and RZ2, respectively. The similarity in the spectral signatures shows the difficulty of mineral detection in those samples. In so many cases, the mean spectral signatures of the classes only differ a scale factor which makes it difficult for the spectral classifier to distinguish the mineral classes due to the spectral similarity. For RZ2, the classification task is even more challenging, since even the scale factors between the spectral signatures from the training set are negligible, e.g., the difference between muscovite-dominated and albite-dominated classes in Figure 9a or albite-dominated and apatite-dominated classes in Figure 9b. Finally, and most importantly, the mean spectral signatures of HC shown in Figure 8e and Figure 9e reveal better disparities of the mineral classes for LWIR for both datasets which could confirm the high accuracies obtained by HC and the importance of this sensor for such a classification task. We should emphasize that the spectral features shown in Figure 8 and Figure 9 are the mean spectral features captured by the sensors over the labeled pixels which were used for training the pixel-wise classifier and they do not necessarily reflect the pure spectral features of the minerals.

### 5.2. Effects of Noise Reduction and Spatial Information Extraction

To further analyze MOSFus, the performances of two simpler versions of the proposed method have been evaluated. In this context, we considered two scenarios: (1) MOSFus${}_{NoHyMiNoR}$, which uses MOSFus without applying the denoising step (i.e., HyMiNoR) prior to the reduction step on the hyperspectral datasets, and (2) MOSFus${}_{NoMP}$, which uses RGB with no MPs. The results are given in Table 6 and Table 7 for the TS4 and RZ2, respectively. The OAs obtained for TS4 and RZ2 were 0.8143 and 0.7187, respectively, without applying HyMiNoR. By comparing these intermediate results with the results of MOSFus (i.e., 0.8957 and 0.8631 for TS4 and RZ2, respectively), we can confirm that applying HyMiNoR leads to considerable improvements for both datasets. The comparisons of OA also show improvements after using MP. MOSFus obtained the overall accuracies of 0.8957 and 0.8631 for TS4 and RZ2, respectively, while MOSFus${}_{NoMP}$ obtained the overall accuracies of 0.8846 and 0.8244 for TS4 and RZ2, respectively. The purpose of MP and HyMiNoR is to improve the classification accuracies of some mineral phases that might otherwise be mislabeled. The highest mineral accuracies in each row are highlighted in the tables. In more detail, for TS4, exploiting HyMiNoR improves the accuracies of all the minerals except muscovite; and for RZ2 it considerably improves calcite-dominated, and degrades the accuracies of apatite and muscovite (the accuracies of albite and goethite-dominated degrade less than 1%). Overall, the improvements achieved by applying MP and HyMiNoR are more consistent for the minerals of TS4 compared to the ones existing in RZ2.

### 5.3. Processing Time

The needed time for the acquisition of the data varies depending on the type of sensor. The measurement of the sample set with the optical hyperspectral sensors above the conveyor belt was in total 30 sec. This includes the acquisition of the samples and calibration panels. For the MLA experiments it took in total six hours to acquire and pre-analyze the data. We needed for the sample preparation for the MLA measurements, in total, one day. For the classification method, the processing time depends on the spatial and spectral size for the unsupervised sections (i.e., denoising (HyMiNoR), MP, and dimensionality reduction (SSLRA)), and for the supervised section, i.e., the SVM, the processing time mainly depends on the number of training samples. For instance, for TS4, the processing times of the MP is 6 sec, for the SVM it is 45 sec, HyMiNoR—96 minutes, and SSLRA—64 minutes. Therefore, the main processing time of the method is related to HyMiNoR and SSLRA which are applied for every hyperspectral dataset. This comparison reveals the advantage of the MOSFus over MLA in terms of processing time.

## 6. Conclusions

In this paper, we propose a multi-optical sensor fusion technique (called MOSFus) for the mineral classification and domain mapping. MOSFus contains four main steps: 1—spatial information extraction from RGB, 2—noise reduction, 3—dimensionality reduction, and 4—supervised classification of the reduced spectral and extracted spatial features. The proposed methodology was evaluated based on two datasets from geologically relevant samples. Both datasets used in the study were acquired by a modular multisensor imaging setup. The results confirm that the proposed approach can considerably outperform the classification accuracies of the other fusion techniques and the individual sensors for both datasets. Additionally, it was demonstrated that MOSFus provides better mapping results than the other techniques used in the experiments for both datasets in terms of extracting the mineral spatial structures. Moreover, the experiments showed the advantage of the LWIR spectral range for the classification of target mineral domains existing in the drill core samples compared with the other spectral ranges (i.e., VNIR and SWIR). Many parts of the proposed framework (e.g., preprocessing, denoising, morphological profiles, and feature extraction-based multisensor data fusion) are unsupervised and can easily be applied to a new sample. The classification step using SVM is, however, supervised and it demands training data to define classification boundaries in the feature space to categorize different classes of interest. This means that if the new sample contains different classes than those used to trained the classifier, this method cannot be directly applied to the new sample. As a result, if the samples contain similar classes this method will be applicable to the new sample. On the other hand, this method can be applied on any geological sample having training sets.

## Figures and Tables

**Table 1:** Specifications and settings used for the hyperspectral sensors and the RGB cameras. The pixel size is expressed in the length of a quadratic pixel.

Sensor	Spectral Range/nm	Image Dimension/px	Pixel Size/mm
RGB	-	4000 × 2000	0.15
FX10	400–1000	1024 per line	0.58
FX17	950–1700	640 per line	0.96
sCMOS	400–1000	2185 per line	0.08
FENIX	380–2500	384 per line	1.54
HC	7700–11800	320 × 256	0.62

**Table 2:** The numbers of training and test samples used for the classification purpose on the TS4 dataset.

Class No.	Class	Training	Testing
1	Gypsum/Anhydrite	200	3258
2	Quartz	200	2808
3	Sulfides	200	2846
4	Muscovite	200	3093
5	Feldspar	200	2859
	Total	1000	14,864

**Table 3:** The classification accuracies of different fusion techniques and different sensors using SVM applied on the TS4 dataset. The number of features used is given in brackets and the highest accuracy in each row is shown in bold.

Class No.	RGB	RGB_MP	sCMOS	FX10	FX17	FENIX	HC	OTVCA_Fus	MOSFus
	(3)	(27)	(650)	(224)	(224)	(623)	(91)	(38)	(77)
1	0.3659	0.4773	0.5862	0.5571	0.6786	0.7017	0.8471	0.7719	**0.9561**
2	0.4886	0.5427	0.635	0.5231	0.5167	0.5231	0.8925	0.8511	**0.8839**
3	0.8363	0.9037	0.7351	0.8078	0.5548	0.7034	0.7765	0.928	**0.9589**
4	0.5021	0.7042	0.8031	0.7685	0.8946	0.711	0.7747	**0.9463**	0.8173
5	0.6184	0.723	0.7272	0.6709	0.6831	0.8349	0.7866	0.7664	**0.8601**
AA	0.5622	0.6702	0.6973	0.6655	0.6656	0.6948	0.8155	0.8527	**0.8953**
OA	0.556	0.6658	0.6962	0.6646	0.6701	0.6958	0.8155	0.852	**0.8957**

**Table 4:** The numbers of training and test samples used for the classification purpose on the RZ2 dataset.

Class No.	Class	Training	Testing
1	Albite	102	780
2	Apatite	104	1717
3	Goethite-dominated	107	104
4	Calcite-dominated	101	88,165
5	Muscovite	102	1967
	Total	516	92,733

**Table 5:** The classification accuracies of different fusion techniques and different sensors using SVM applied on the RZ2 dataset. The number of features used is given in brackets and the highest accuracy in each row is shown in bold.

Class No.	RGB	RGB_MP	sCMOS	FX10	FX17	FENIX	HC	OTVCA_Fus	MOSFus
	(3)	(27)	(480)	(224)	(224)	(623)	(91)	(38)	(77)
1	0.5026	0.5679	0.5718	0.8449	0.9051	0.8154	0.8538	0.8713	**0.8974**
2	0.0856	0.2685	0.3873	0.7152	0.7758	0.6476	0.7472	**0.8096**	0.7554
3	0.7981	0.6635	0.8269	0.9808	0.9808	0.9135	**1**	0.97	0.9904
4	0.2943	0.3311	0.3861	0.6047	0.53	0.791	0.8268	0.7787	**0.8669**
5	0.1149	0.4398	0.4281	0.6802	0.7661	0.7229	0.7067	0.7402	**0.7672**
AA	0.3591	0.4541	0.52	0.7652	0.7916	0.7781	0.8269	0.834	**0.8555**
OA	0.289	0.3346	0.389	0.6108	0.5432	0.7872	0.8232	0.7794	**0.8631**

**Table 6:** The effects of HyMiNoR and morphological profile (MP) on the classification accuracies for the TS4 dataset. The highest accuracy in each row is shown in bold.

			MOSFus*_NoMP_*	MOSFus*_NoHyMiNoR_*	MOSFus
1	Gypsum/Anhydrite		**0.9616**	0.6621	0.9561
2	Quartz		0.875	0.7304	**0.8839**
3	Sulides		0.9519	0.916	**0.9589**
4	Muscovite		0.7873	**0.9602**	0.8173
5	Feldspar		0.8444	0.8111	**0.8601**
AA	–		0.884	0.816	**0.8953**
OA	–		0.8846	0.8143	**0.8957**

**Table 7:** The effects of HyMiNoR and MP on the classification accuracies for the RZ2 dataset. The highest accuracy in each row is shown in bold.

			MOSFus*_NoMP_*	MOSFus*_NoHyMiNoR_*	MOSFus
1	Albite		**0.9051**	**0.9051**	0.8974
2	Apatite		0.7764	**0.8346**	0.7554
3	Goethite-dominated		0.9904	**1**	0.9904
4	Calcite-dominated		0.8253	0.7122	**0.8669**
5	Muscovite		0.7855	**0.8216**	0.7672
AA	–		**0.8565**	0.8547	0.8555
OA	–		0.8244	0.7187	**0.8631**
